# The expression landscape of *JAK1* and its potential as a biomarker for prognosis and immune infiltrates in NSCLC

**DOI:** 10.1186/s12859-021-04379-y

**Published:** 2021-09-29

**Authors:** Kaikai Shen, Yuqing Wei, Tangfeng Lv, Yong Song, Xiaogan Jiang, Zhiwei Lu, Ping Zhan, Xianghai Wang, Meng Fan, Weihua Lu

**Affiliations:** 1 Department of Critical Care Medicine, The First Affiliated Hospital of Wannan Medical College (Yijishan Hospital of Wannan Medical College), Wuhu, 241000 China; 2 Department of Respiratory Medicine, The First Affiliated Hospital of Wannan Medical College (Yijishan Hospital of Wannan Medical College), Wuhu, 241000 China; 3 Department of Respiratory Medicine, Jinling Hospital, Nanjing University School of Medicine, Nanjing, 210002 China; 4 Department of Radiology Medicine, The No. 2 People’s Hospital, Hefei, 230000 China

**Keywords:** *JAK1*, Immune infiltrating, Prognosis, NSCLC

## Abstract

**Background:**

Janus-activated kinase-1 (*JAK1*) plays a crucial role in many aspects of cell proliferation, differentiation, apoptosis and immune regulation. However, correlations of *JAK1* with prognosis and immune infiltration in NSCLC have not been documented.

**Methods:**

We analyzed the relationship between *JAK1* expression and NSCLC prognosis and immune infiltration using multiple public databases.

**Results:**

*JAK1* expression was significantly decreased in NSCLC compared with that in paired normal tissues. *JAK1* overexpression indicated a favourable prognosis in NSCLC. In subgroup analysis, high *JAK1* expression was associated with a preferable prognosis in lung adenocarcinoma (OS: HR, 0.74, 95% CI from 0.58 to 0.95, log-rank *P* = 0.017), not squamous cell carcinoma. In addition, data from Kaplan–Meier plotter revealed that *JAK1* overexpression was associated with a preferable prognosis in male and stage N2 patients and patients without distant metastasis. Notably, increased levels of *JAK1* expression were associated with an undesirable prognosis in patients with stage 1 (OS: HR, 1.46, 95% CI from 1.06 to 2.00, *P* = 0.02) and without lymph node metastasis (PFS: HR, 2.18, 95% CI from 1.06 to 4.46, *P* = 0.029), which suggests that early-stage NSCLC patients with *JAK1* overexpression may have a bleak prognosis. Moreover, multiple immune infiltration cells, including NK cells, CD8 + T and CD4 + T cells, B cells, macrophages, neutrophils, and dendritic cells (DCs), in NSCLC were positively correlated with *JAK1* expression. Furthermore, diverse immune markers are associated with *JAK1* expression.

**Conclusions:**

*JAK1* overexpression exhibited superior prognosis and immune infiltration in NSCLC.

**Supplementary Information:**

The online version contains supplementary material available at 10.1186/s12859-021-04379-y.

## Introduction

Lung cancer, as a malignant tumour with high morbidity and mortality, poses a serious threat to people’s physical and mental health [1]. Non-small-cell lung cancer (NSCLC) accounts for approximately 80% of all lung cancer cases [2]. With the advent of precision therapy, lung cancer treatment has entered molecular therapy, including targeted therapy, anti-angiogenesis therapy and immunotherapy [3]. However, the prognosis has not improved significantly, and the 5-year survival rate remains poor [1]. In recent years, clinical studies have shown that immunotherapy (*PD-1*/*L1* monoclonal antibody, *CTLA-4* inhibitor) has great potential in the treatment of lung cancer patients without epidermal growth factor receptor (*EGFR*) and anaplastic lymphoma kinase (*ALK*) mutations [4]. Nevertheless, immunotherapy only activates immune cells in a subset of patients. With the continuous exploration of the tumour immune microenvironment (TME), which can directly or indirectly affect the development of tumours, including promoting tumour angiogenesis, changing the biological characteristics of the tumour, promoting immune escape, and even regulating the activity of cancer stem cells (CSCs) [5, 6]. Many studies have found that TAMs (tumour-associated macrophages), TILs (tumour infiltrating lymphocytes) and TINs (tumour-infiltrating neutrophils) in the TME can affect the efficacy of immunotherapy [7, 8]. Hence, it is imperative to find immune infiltration-related biomarkers that are related to the prognosis of NSCLC.

Janus-activated kinase (*JAK*) is an inactive tyrosine protein kinase that consists of four family members, including *JAK1*, *JAK2*, *TYK2*, and *JAK3* [9]. *JAKs* approach each other and are activated by interactive tyrosine phosphorylation, ultimately leading to signal transducer and activator of transcription (*STAT*) proteins forming a homo/heterodimer that is incorporated into the nucleus and binding to the target gene promoter to activate transcription and expression [10]. Previous studies have shown that the *JAK1*/*STAT3* pathway is widely involved in many significant biological processes, such as cell proliferation, differentiation, apoptosis and immune regulation [11–13]. *JAK* family kinases play an essential role in cytokine signalling. Functionally acquired *JAK1* mutations can encourage the development of cancers, especially leukaemia. Abnormal *JAK1* expression either promotes or suppresses tumour growth [10, 14, 15]. Chen et al. [10] showed that high expression of *JAK1* mRNA was associated with TNM (Tumor, Node, Metastasis) stage and superior prognosis of breast cancer. In addition, infiltration and enrichment of immunoregulatory cells were significantly positively correlated with *JAK1* expression. In contrast, Zhang et al. [16] showed that *JAK1* signal activation could promote the proliferation of bladder cancer cells and lead to a poor prognosis. Hu et al. also indicated that *JAK1/STAT3* plays a crucial role in ovarian cancer as a pro-oncogenic signalling pathway [17]. Whether *JAK1* expression is involved in the prognosis and the level of immune infiltration in NSCLC still needs to be further explored.

In our descriptive study, we explored the expression landscape of *JAK1* in NSCLC and its relationship with prognosis using shared databases, including TIMER, GEPIA, Kaplan–Meier Plotter and PrognoScan. We also visualized the relationship between *JAK1* and immune infiltration using TIMER and TISIDB. Moreover, correlations between *JAK1* expression and multiple gene marker sets related to immune infiltrates were also analysed via TIMER and GEPIA.

## Materials and methods

### TIMER database analysis

The TIMER (Tumour Immune Estimation Resource) web server is a comprehensive resource for the systematic analysis of immune infiltrates across diverse cancer types. (https://cistrome.shinyapps.io/timer/) [18]. The abundances of six immune infiltrates (B cells, CD4^+^ T cells, CD8^+^ T cells, neutrophils, macrophages, and dendritic cells) were estimated by the TIMER algorithm. We evaluated the correlation between *JAK1* expression levels and various immune infiltrating cells via the TIMER algorithm. In addition, *JAK1* expression profiles across various tumour samples and paired normal tissues from the TCGA data in TIMER were also determined. Finally, to further identify other potential subtypes of immune cell infiltration, we also analysed the correlation between *JAK1* expression and diverse immune cell markers, including monocytes, tumour-associated macrophages (TAMs), M1 macrophages, M2 macrophages, CD8^+^ T cells, B cells, neutrophils, dendritic cells, natural killer (NK) cells, T-helper 1 (Th1) cells, T-helper 2 (Th2) cells, Tregs and exhausted T cells (https://www.rndsystems.com/cn/resources/cell-markers/immune-cells). Tumour purity was also determined. The gene expression level was described in terms of log_2_TAM. *JAK1* expression was drawn in the x-axis, while marker genes were drawn in the y-axis. A scatterplot was used to describe the specific connection between every immune gene marker and *JAK1* expression.

### TISIDB analysis

TISIDB is also a web portal for tumour and immune system interaction, which integrates multiple heterogeneous data types. (http://cis.hku.hk/TISIDB/index.php) [19]. We explored the correlation between *JAK1* expression in NSCLC and the abundance of multiple immune cells, including activated CD4 T cells (Act_CD4), activated dendritic cells (Act_DCs), immature dendritic cells (iDCs), neutrophils, natural killer cells (NKs), plasmacytoid dendritic cells (pDCs), central memory CD4 cells (Tcm_CD4), and effector memory CD8 cells (Tem_CD8). The relative abundance of each immune cell was inferred by using gene set variation analysis (GSVA) based on the gene expression profile. *JAK1* expression was drawn on the x-axis, while the abundance of immune cells was drawn on the y-axis. A scatterplot was used to display the correlation between the abundance of each immune cell and *JAK1* expression.

### GEPIA database analysis

To further verify the gene marker associated with immune infiltration in NSCLC. We used the public database Gene Expression Profiling Interactive Analysis (GEPIA) (http://gepia.cancer-pku.cn/index.html) [20], which analyses the RNA sequencing expression from the TCGA and GTEx projects of 9736 tumours and 8587 normal samples. The correlation coefficient was determined by the Spearman method. The tumour and normal tissue datasets were used for analysis. *JAK1* expression profiles across LUAD (lung adenocarcinoma) and LUSC (lung squamous cell carcinoma) samples and paired normal tissues from GEPIA were also analysed.

### Prognostic analysis

We used public databases including Kaplan–Meier Plotter (https://kmplot.com/analysis/) [21] and PrognoScan (http://dna00.bio.kyutech.ac.jp/PrognoScan/index.html) [22] to examine the relationship between *JAK1* expression level and NSCLC prognosis. The Kaplan–Meier plotter is competent for assessing the effect of 54,000 genes on prognosis in 21 cancer types. Sources included the GEO, EGA, and TCGA databases. The hazard ratio (HR) and its 95% confidence interval (95% CI) for OS (overall survival) and PFS (progression-free survival) in NSCLC were calculated. The log-rank *P* value was likewise computed.

Similarly, the prognostic database PrognoScan was designed to analyse the correlation between JAK1 expression and overall survival (OS). The threshold was set as a Cox *P* value < 0.05.

### Statistical analysis

The results examined in TIMER and GEPIA are displayed with *P* values determined by *t* tests, fold changes, and gene ranks. Survival outcomes were presented with Kaplan–Meier plots and PrognoScan, and the results are displayed with HR and Cox *P* values from a log-rank test. The correlation between *JAK1* expression and each gene marker was assessed by Spearman’s correlation test and statistical significance. The strength of the correlation was defined as follows: 0.00–0.19 “very weak”, 0.20–0.39 “weak”, 0.40–0.59 “moderate”, 0.60–0.79 “strong”, and 0.80–1.0 “very strong”. For all analyses, a *P* value less than 0.05 indicates statistical significance.

## Results

### *JAK1* expression in multiple human tumours

**Fig. 1:**
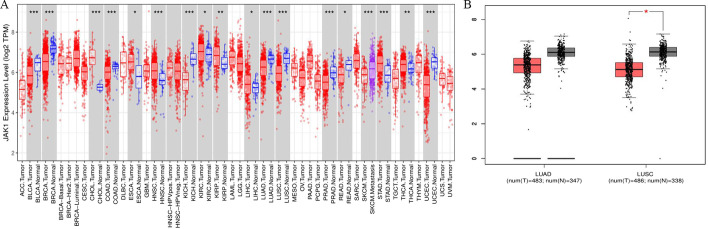
*JAK1* expression profile in various types of human tumours. **A** Human *JAK1* expression profile across various tumour samples and paired normal tissues from the TCGA data in TIMER. **B** Human *JAK1* expression profile in NSCLC from the GEPIA database. The ordinate of **B** indicates the expression level of *JAK1* (log_2_ TPM). *LUAD* lung adenocarcinoma, *LUSC* lung squamous cell carcinoma, *NSCLC* non-small-cell lung cancer, *TPM* transcripts per million, *N* normal, *T* tumour. ^*^P < 0.05, ^**^P < 0.01, ^***^P < 0.001

We evaluated the differences in *JAK1* expression in various human tumour tissues and paired normal tissues using RNA sequencing data from the TCGA. The detailed expression of *JAK1* in the tumour and adjacent tissues is shown in Fig. 1A. *JAK1* expression was significantly decreased in BLCA (bladder urothelial carcinoma), BRCA (breast invasive carcinoma), COAD (colon adenocarcinoma), KICH (kidney chromophobe), LUAD, LUSC, PRAD (prostate adenocarcinoma), READ (rectum adenocarcinoma), and UCEC (uterine corpus endometrial carcinoma) compared to that in adjacent normal tissues, while the expression of *JAK1* was significantly higher in CHOL (cholangiocarcinoma), ESCA (oesophageal carcinoma), HNSC (head and neck squamous cell carcinoma), KIRC (kidney renal clear cell carcinoma), KIRP (kidney renal papillary cell carcinoma), LIHC (liver hepatocellular carcinoma), STAD (stomach adenocarcinoma), and THCA (thyroid carcinoma) than that in adjacent normal tissues.

To further evaluate the expression patterns of *JAK1* in NSCLC, the GEPIA database was further selected. Similar results were likewise obtained, namely, *JAK1* expression in LUAD and LUSC was significantly lower than that in the paired normal tissues (Fig. 1B).

### *JAK1* expression predicts the prognosis of NSCLC

**Fig. 2:**
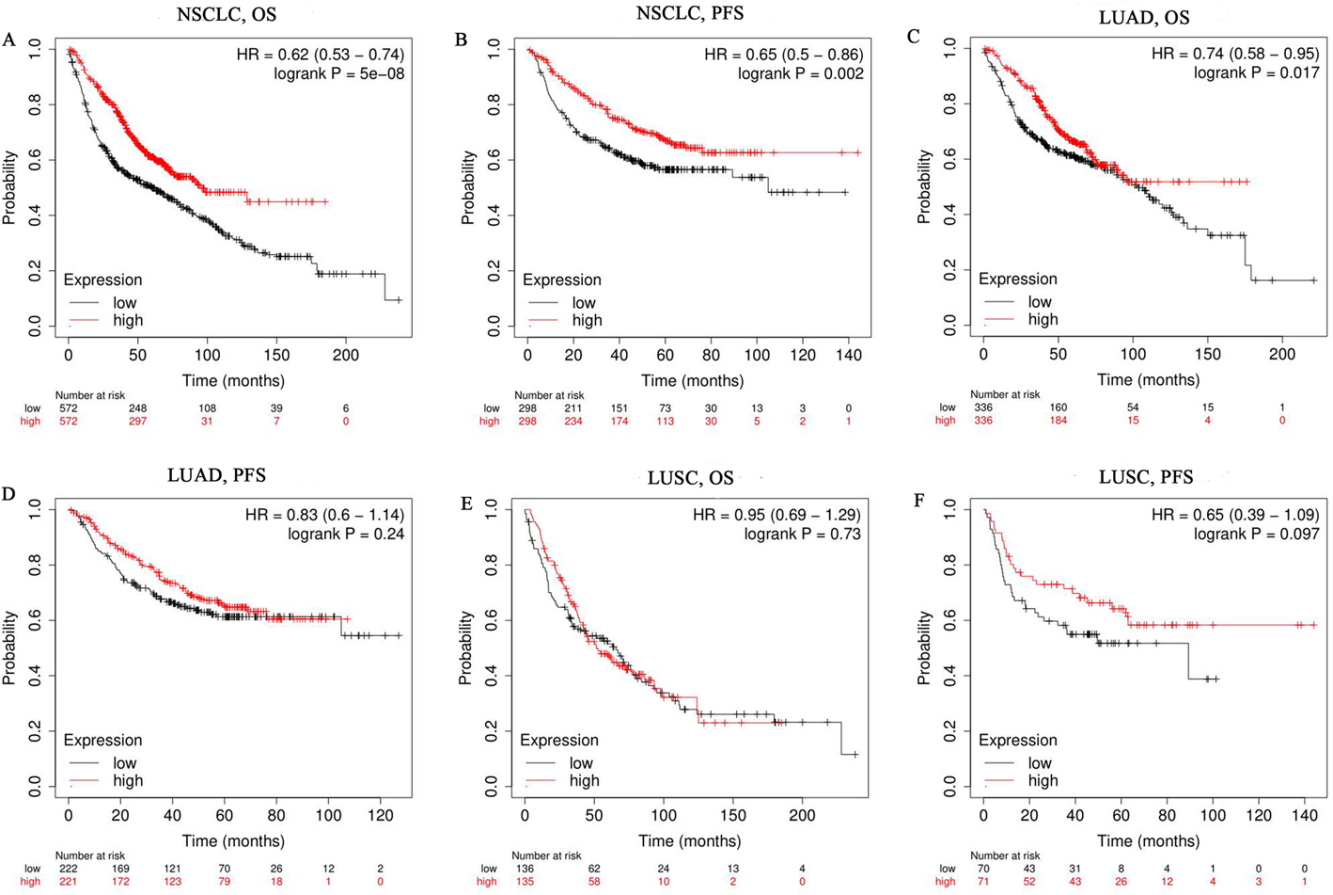
Kaplan–Meier survival curves comparing high and low expression of *JAK1* in NSCLC. **A**, **B** OS and PFS survival curves of NSCLC (n = 1144, n = 596). **C**, **D** OS and PFS survival curves of LUAD (n = 672, n = 443). **E**, **F** OS and PFS survival curves of LUSC (n = 271, n = 141). *OS* overall survival, *PFS* progression-free survival, *LUAD* lung adenocarcinoma, *LUSC* lung squamous cell carcinoma, *NSCLC* non-small-cell lung cancer, *HR* hazard ratio

Next, we explored the prognostic value of *JAK1* for NSCLC by adopting two public databases. First, we investigated *JAK1* expression and the prognosis of NSCLC, LUAD and LUSC using Kaplan–Meier Plotter, which principally focused on the strength of the information from the GEO, EGA and TCGA miRNA gene chips. The results showed that high *JAK1* expression indicated a favourable prognosis in NSCLC (OS: HR, 0.62, 95% CI from 0.53 to 0.74, log-rank *P* < 0.001; PFS: HR, 0.65, 95% CI from 0.50 to 0.86, log-rank *P* = 0.002). In the subgroup analysis, the high expression of *JAK1* in LUAD lasted longer in OS (HR: 0.74, 95% CI from 0.58 to 0.95, log-rank *P* = 0.017), but there was no benefit in PFS (HR: 0.83, 95% CI from 0.60 to 1.14, log-rank *P* = 0.24). In LUSC, high expression of *JAK1* was associated with longer duration of PFS (HR: 0.65, 95% CI from 0.39 to 1.09, log-rank *P* = 0.097), while the difference was not statistically significant. In addition, there was no benefit in OS (HR: 0.95, 95% CI from 0.69 to 1.29, log-rank *P* = 0.73). (Fig. 2).

Next, we investigated the association of *JAK1* expression and prognosis with distinct clinicopathological features in NSCLC (Table 1). *JAK1* overexpression related to superior OS and PFS in males (HR: 0.64, 0.62, 95% CI from 0.52 to 0.79, *P* < 0.001) rather than females. In addition, the higher expression of *JAK1* is associated with preferable OS in patients with N2 lymph node metastasis (HR: 0.39, 95% CI from 0.17 to 0.86, *P* = 0.016) without distant metastasis (HR: 0.73, 95% CI from 0.56 to 0.93, *P* = 0.013) of NSCLC. Notably, overexpression of *JAK1* is associated with undesirable prognosis in patients with stage 1 NSCLC (OS: HR, 1.46, 95% CI from 1.06 to 2.00, *P* = 0.02) and without lymph node metastasis (PFS: HR, 2.18, 95% CI from 1.06 to 4.46, *P* = 0.029), which implicit early NSCLC patients with *JAK1* overexpression may have a poor prognosis. Regrettably, there were no statistically significant differences between *JAK1* expression and prognosis in females, stage 2 to 3, stage T1 to T4, N1 lymph node metastasis or prior chemotherapy. The exact survival time is shown in Additional file 1: Table S1.

**Table 1:** Association between *JAK1* expression and prognosis with different clinicopathological features of NSCLC by Kaplan–Meier plotter

Clinicopathological characteristics	Overall survival (n = 1144)			Progression-free survival (n = 596)		
	N	HR	***P*** value	N	HR	***P*** value
Gender						
Male	659	0.64 (0.52–0.79)	2.20E−05	343	0.61 (0.43–0.86)	0.0043
Female	374	0.86 (0.61–1.21)	0.38	253	0.77 (0.49–1.20)	0.25
Stage						
1	449	1.46 (1.06–2.00)	0.02	596	1.26 (0.81–1.95)	0.31
2	161	0.99 (0.63–1.55)	0.95	125	0.64 (0.38–1.11)	0.108
3	44	1.11 (0.55–2.25)	0.77	17	–	–
AJCC stage T						
1	224	0.86 (0.57–1.28)	0.44	54	0.95 (0.23–3.53)	0.94
2	190	0.86 (0.58–1.27)	0.44	121	1.75 (0.94–3.27)	0.073
3	29	0.83 (0.38–1.85)	0.65	2	–	–
4	23	0.74 (0.30–1.84)	0.52	0	–	–
AJCC stage N						
0	324	0.87 (0.63–1.19)	0.38	126	2.18 (1.06–4.46)	0.029
1	102	0.64 (0.38–1.08)	0.09	51	0.98 (0.40–2.41)	0.96
2	32	0.39 (0.17–0.86)	0.016	0	–	–
AJCC stage M						
0	462	0.73 (0.56–0.93)	0.013	177	1.66 (0.95–2.9)	0.07
Smoking history						
Yes	300	1.59 (1.04–2.24)	0.029	297	0.73 (0.48–1.08)	0.11
No	141	2.24 (0.96–5.25)	0.056	141	1.39 (0.75–2.58)	0.29
Chemotherapy						
Yes	34	0.82 (0.26–2.64)	0.74	34	1.18 (0.45–3.11)	0.74
No	21	0.18 (0.02–1.52)	0.075	21	0.63 (0.19–2.07)	0.44

*N* Number, *HR* Hazard Ratio

Finally, we selected the PrognoScan database to further verify the relationship between *JAK1* expression and prognosis in NSCLC. Five cohorts containing a total of 530 patients with NSCLC and LUAD showed that high expression of *JAK1* was associated with favourable OS (Table 2).

**Table 2:** Survival analysis of *JAK1* mRNA in NSCLC from the PrognoScan database

Dataset	Subtype	Endpoint	Number	Ln (HR-high/HR-low)	COX ***P*** value	ln HR	HR [95% CIlow–CIup]
jacob-00182-CANDF	LUAD	OS	82	− 1.11	0.002460	− 1.37	0.25 [0.10–0.62]
GSE31210	LUAD	OS	204	− 1.18	0.026306	− 1.13	0.32 [0.12–0.88]
GSE11117	NSCLC	OS	41	− 1.55	0.034669	− 0.78	0.46 [0.22–0.95]
MICHIGAN-LC	LUAD	OS	86	− 1.25	0.094138	− 0.85	0.43 [0.16–1.16]
GSE13213	LUAD	OS	117	− 0.73	0.057336	− 0.43	0.65 [0.42–1.01]

### Correlation of JAK1 expression and immune infiltration

**Fig. 3:**
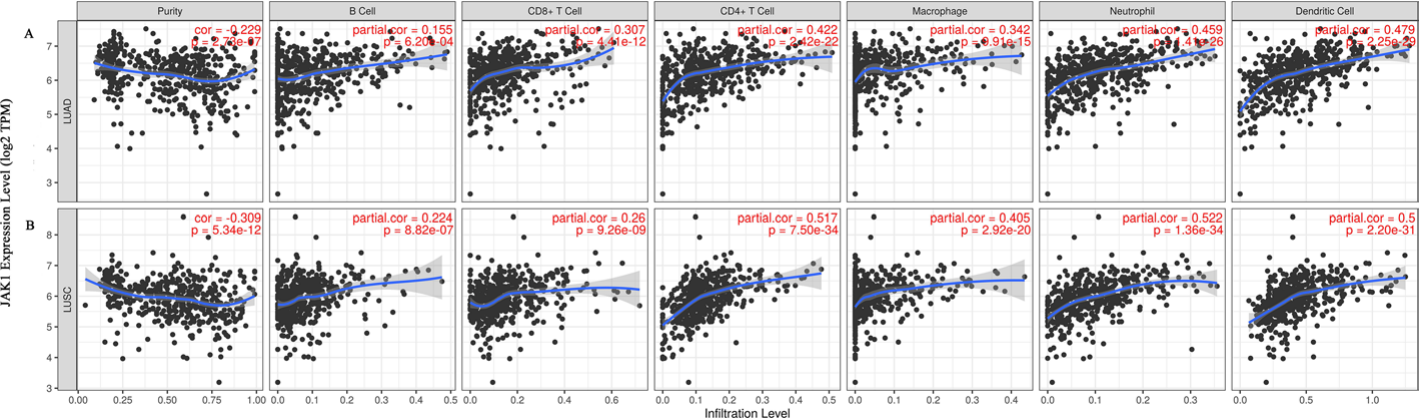
TIMER database showing the relationship between *JAK1* expression level and immune infiltration in LUAD and LUSC. **A** *JAK1* expression was negatively correlated with tumour purity and significantly positively correlated with infiltrating levels of B cells, CD8^+^ T cells, CD4^+^ T cells, macrophages, neutrophils, and dendritic cells in LUAD. **B** *JAK1* expression was negatively correlated with tumour purity and significantly positively correlated with infiltrating levels of B cells, CD8^+^ T cells, CD4^+^ T cells, macrophages, neutrophils, and dendritic cells in LUSC. *LUAD* lung adenocarcinoma, *LUSC* lung squamous cell carcinoma. A *P* value less than 0.05 indicated statistical significance

Tumour infiltrating lymphocytes (TILs) are closely related to prognosis and subsequent immunotherapy in lung cancer [23, 24]. We investigated the correlation between *JAK1* expression level and immune cell infiltration in LUAD and LUSC from TIMER. The results showed that *JAK1* expression was negatively correlated with tumour purity (*r* = − 0.229, *P* = 2.73e-07) and significantly positively correlated with infiltrating levels of B cells (*r* = 0.155, *P* = 6.20e−04), CD8^+^ T cells (*r* = 0.307, *P* = 4.41e−12), CD4^+^ T cells (*r* = 0.422, *P* = 2.42e−22), macrophages (*r* = 0.342, *P* = 9.91e−15), neutrophils (*r* = 0.459, *P* = 1.41e−26), and dendritic cells (*r* = 0.479, *P* = 2.25e−29). Similar results were also observed in LUSC. *JAK1* expression was negatively correlated with tumour purity (*r* = − 0.309, *P* = 5.34e−12) and significantly positively correlated with infiltrating levels of B cells (*r* = 0.224, *P* = 8.82e−07), CD8^+^ T cells (*r* = 0.26, *P* = 9.26e−09), CD4^+^ T cells (*r* = 0.517, *P* = 7.50e−34), macrophages (*r* = 0.405, *P* = 2.92e−20), neutrophils (*r* = 0.522, *P* = 1.36e−34), and dendritic cells (*r* = 0.5, *P* = 2.20e−31) (Fig. 3).

**Fig. 4:**
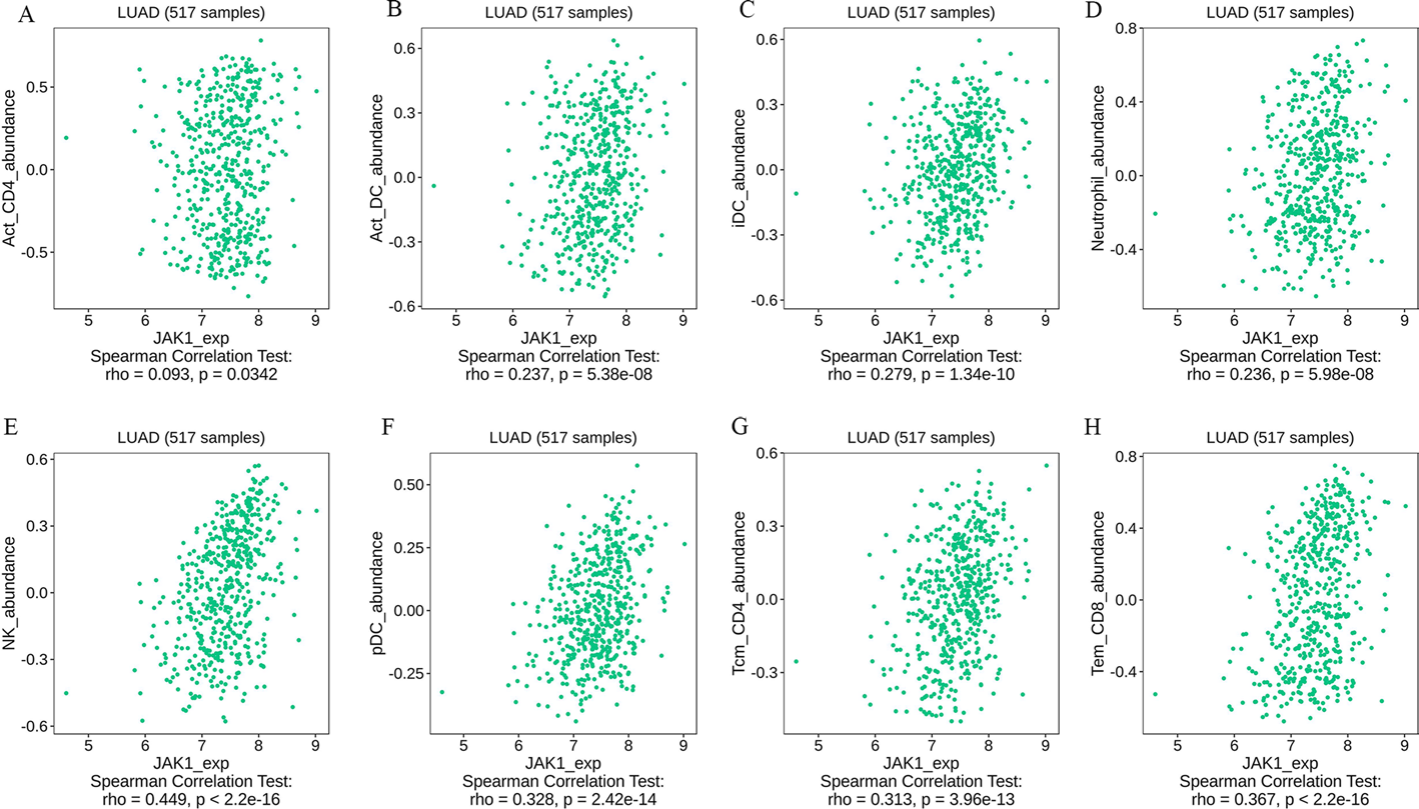
Association between *JAK1* expression level and immune cell infiltration in LUAD from the TISIDB web portal (517 samples). **A**–**H** *JAK1* expression was significantly positively correlated with infiltrating levels of Act_CD4, Act_DCs, iDCs, neutrophils, NK cells, pDCs, Tcm_CD4 and Tem_CD8. *LUAD* lung adenocarcinoma, *Act_CD4* activated CD4 T cells, *Act_DCs* activated dendritic cells, *iDCs* immature dendritic cells, *NK cells* natural killer cells, *pDCs* plasmacytoid dendritic cells, *Tcm_CD4* central memory CD4 cells, *Tem_CD8* effector memory CD8 cells. A *P* value less than 0.05 indicated statistical significance

In addition, the public database TISIDB also explored the correlation between the abundance of multiple immune cells and *JAK1* expression in NSCLC. The enrichment of diversified immune cells, such as Act_CD4, Act_DCs, iDCs, neutrophils, NK cells, pDCs, Tcm_CD4 and Tem_CD8, was positively correlated with *JAK1* expression in LUAD and LUSC. What needs illustration is that *JAK1* expression has no significant corrections with infiltrating levels of Act_CD4 in LUSC. For details, please refer to Fig. 4 and Additional file 1: Fig S1.

### Correlations between *JAK1* expression and immune gene markers

To further understand the interaction between *JAK1* expression and TME in NSCLC. We further explored the potential correlation between *JAK1* and immune gene markers in the public databases TIMER and GEPIA (Tables 3, 4). These gene markers depicted diverse immune infiltration cells, including monocytes, TAMs, M1 macrophages, M2 macrophages, CD8^+^ T cells, B cells, neutrophils, dendritic cells and NK cells. In addition, various T cells, including Th1, Th2, Tregs, and T cell exhaustion, which play different functions in the TME, were included. Although they were adjusted for tumour purity, most immune markers remained significantly related to *JAK1* expression levels in LUAD and LUSC.

**Table 3:** Correlation analysis between *JAK1* and diverse immune gene markers in LUAD and LUSC from the TIMER database

Description	Gene markers	LUAD				LUSC			
		None		Purity		None		Purity	
		Cor	***P***	Cor	***P***	Cor	***P***	Cor	***P***
Monocyte	CD14	0.262	***	0.2	***	0.425	***	0.323	***
	CSF1R	0.503	***	0.463	***	0.553	***	0.482	***
	CD86	0.395	***	0.344	***	0.444	***	0.349	***
TAM	CCL2	0.254	***	0.19	***	0.368	***	0.296	***
	CD80	0.385	***	0.331	***	0.383	***	0.294	***
	CD68	0.383	***	0.339	***	0.382	***	0.29	***
M1	IRF5	0.323	***	0.277	***	0.068	0.126	0.049	0.281
	NOS2	0.22	***	0.194	***	0.062	0.165	0.071	0.122
M2	CD163	0.45	***	0.414	***	0.458	***	0.378	***
	ARG1	0.113	*	0.11	0.015	− 0.069	0.125	− 0.074	0.107
	MS4A4A	0.352	***	0.3	***	0.345	***	0.24	***
CD8^+^ T cell	CD8A	0.248	***	0.177	***	0.262	***	0.175	**
	CD8B	0.118	*	0.051	0.257	0.188	***	0.135	*
B cell	CD19	0.121	*	0.02	0.653	0.277	***	0.146	*
	CD79A	0.137	*	0.047	0.295	0.323	***	0.198	***
Neutrophils	CEACAM8	0.259	***	0.252	***	0.072	0.106	0.048	0.293
	MPO	0.22	***	0.183	***	0.342	***	0.29	***
	CCR7	0.361	***	0.295	***	0.39	***	0.291	***
	CD11b(ITGAM)	0.472	***	0.435	***	0.578	***	0.517	***
Dendritic cell	HLA-DPB1	0.313	***	0.249	***	0.457	***	0.371	***
	HLA-DQB1	0.253	***	0.192	***	0.328	***	0.245	***
	HLA-DRA	0.281	***	0.215	***	0.387	***	0.292	***
	HLA-DPA1	0.353	***	0.3	***	0.454	***	0.371	***
	BDCA-1(CD1C)	0.294	***	0.243	***	0.341	***	0.227	***
	BDCA-4(NRP1)	0.386	***	0.377	***	0.523	***	0.473	***
	CD11C(ITGAX)	0.383	***	0.327	***	0.513	***	0.427	***
	CD141(THBD)	0.366	***	0.344	***	0.032	0.469	− 0.006	0.89
NK cell	KIR2DL1	0.046	0.296	0.023	0.612	0.143	*	0.091	0.047
	KIR2DL3	0.104	0.019	0.06	0.187	0.158	**	0.117	0.010
	KIR2DL4	0.062	0.163	0.015	0.737	0.113	0.011	0.051	0.265
	KIR3DL1	0.064	0.15	0.024	0.601	0.241	***	0.187	***
	KIR3DL2	0.139	*	0.087	0.053	0.141	*	0.069	0.134
	KIR3DL3	0.002	0.964	− 0.019	0.67	0.029	0.52	0.002	0.962
	KIR2DS4	0.119	*	0.082	0.068	0.122	*	0.093	0.043
	CD7	0.168	**	0.091	0.043	0.339	***	0.237	***
	XCL1	0.036	0.416	0.004	0.931	− 0.015	0.742	0.017	0.705
Th1	T-bet (TBX21)	0.322	***	0.263	***	0.363	***	0.273	***
	STAT4	0.336	***	0.274	***	0.49	***	0.415	***
	STAT1	0.398	***	0.364	***	0.298	***	0.251	***
	IFN-γ (IFNG)	0.129	*	0.06	0.181	0.121	*	0.057	0.216
	TNF-α (TNF)	0.271	***	0.229	***	0.402	***	0.345	***
Th2	GATA3	0.435	***	0.387	***	0.551	***	0.517	***
	STAT6	0.293	***	0.318	***	0.265	***	0.284	***
	STAT5A	0.53	***	0.495	***	0.549	***	0.491	***
	IL13	0.079	0.072	0.032	0.479	0.152	**	0.083	0.072
Treg	FOXP3	0.354	***	0.297	***	0.481	***	0.402	***
	STAT5B	0.46	***	0.45	***	0.274	***	0.288	***
	TGFβ (TGFB1)	0.487	***	0.451	***	0.312	***	0.266	***
	CCR8	0.422	***	0.378	***	0.442	***	0.358	***
	CD25(IL2RA)	0.357	***	0.305	***	0.368	***	0.273	***
T cell exhaustion	PD-1(PDCD1)	0.225	***	0.144	*	0.357	***	0.267	***
	CTLA4	0.274	***	0.197	***	0.331	***	0.221	***
	LAG3	0.173	***	0.104	0.021	0.257	***	0.181	***
	TIM-3 (HAVCR2)	0.371	***	0.314	***	0.391	***	0.292	***

*LUAD* lung adenocarcinoma, *LUSC* lung squamous cell carcinoma, *TAM* tumour-associated macrophage, *M1* M1 macrophage, *M2* M2 macrophage, *Th* T helper cell, *Treg* regulatory T cell, *Cor* R value of Spearman’s correlation, *None* correlation without adjustment, *Purity* correlation adjusted by purity

*P < 0.01; **P < 0.001; ***P < 0.0001

**Table 4:** Correlation analysis between *JAK1* and relevant genes and markers of monocytes and macrophages in GEPIA

Description	Gene markers	LUAD				LUSC			
		Tumor		Normal		Tumor		Normal	
		R	***P***	R	***P***	R	***P***	R	***P***
Monocyte	CD14	0.3	***	− 0.051	0.7	0.27	***	0.027	0.85
	CD115	0.51	***	0.2	0.13	0.43	***	0.32	0.024
	CD86	0.4	***	− 0.22	0.093	0.28	***	− 0.035	0.81
TAM	CCL2	0.23	***	0.16	0.24	0.23	***	− 0.02	0.89
	CD80	0.35	***	0.21	0.11	0.21	***	0.22	0.13
	CD68	0.4	***	0.01	0.94	0.28	***	0.14	0.32
M1	IRF5	0.25	***	− 0.1	0.43	0.0053	0.91	0.11	0.45
	ROS	0.2	***	0.51	***	0.31	***	0.34	0.015
	NOS2	0.0033	0.94	0.56	***	− 0.042	0.36	0.49	**
M2	CD163	0.33	***	− 0.18	0.18	0.29	***	0.074	0.61
	ARG1	0.05	0.27	0.27	0.036	− 0.027	0.55	0.14	0.34
	MS4A4A	0.33	***	− 0.29	0.024	0.22	***	− 0.038	0.79

*LUAD* lung adenocarcinoma, *LUSC* lung squamous cell carcinoma, *TAM* tumour-associated macrophage, *M1* M1 macrophage, *M2* M2 macrophage

*P < 0.01; **P < 0.001; ***P < 0.0001

Interestingly, the results from TIMER and GEPIA showed that most gene sets of monocytes, M1 macrophages, and TAMs were significantly associated with *JAK1* expression levels in LUAD. However, we discovered that *JAK1* expression was also associated with most gene sets of monocytes and TAMs rather than M1 macrophages. Notably, the majority chemokine ligand, which induced cells of the immune system to enter the site of infection, *CCL-2*, *CD80* and *CD68* of TAMs, *IRF5* and *NOS2* of M1, *CD163* and *MS4A4A* of M2 were strongly related to *JAK1* expression in LUAD (all *P* value < 0.0001). These consequences suggest that *JAK1* may play a vital role in the TME by regulating the function of macrophages. In addition, some of the gene markers, such as *MPO*, *CCR7* and *CD11b* (*ITGAM*), of neutrophils and *CD8A* of CD8^+^ T cells were associated with *JAK1* expression in LUAD and LUSC.

Moreover, the vast majority of gene sets of dendritic cells, including *HLA-DPB1*, *HLA-DQB1*, *HLA-DRA*, *HLA-DPA1*, *BDCA-1*, *BDCA-4* and *CD11C*, were positively correlated with *JAK1* expression levels in LUAD and LUSC. These results indicated that *LAYN* may regulate DCs to play a major role in the TME. Regretfully, nearly all of the gene markers of NK cells had no correlation with *JAK1* expression levels. Furthermore, we investigated the relationship between *JAK1* expression and gene sets of Tregs and T cell exhaustion. All gene sets suggested a positive correlation with *JAK1* expression. Finally, immune checkpoints such as *PD-1*, *CTLA4*, *LAG3* and *TIM3* were strongly connected with the level of *JAK1* expression, which suggested that *JAK1* may play a role in immunotherapy for NSCLC. Further molecular biology experiment verification is needed.

## Discussion

The *JAK1/STAT* signalling pathway, as a stimulant that is intimately related to the physiological function of interferon, plays a significant role in cell growth, differentiation, immune regulation and other aspects [11, 25, 26]. The exhaustive function of *JAK1* in NSCLC has not yet been clarified. Here, we report the expression profile of *JAK1* and its association with prognosis and immune infiltration in NSCLC. We found that *JAK1* was expressed at low levels in NSCLC, and its expression level was positively correlated with the prognosis of NSCLC, especially in LUAD. Interestingly, *JAK1* overexpression was associated with preferable survival in males, stage N2 patients and patients without distant metastasis. In addition, increased levels of *JAK1* expression are associated with undesirable survival in patients with earlier stages (stage 1 and N0), suggesting that early-stage NSCLC patients with *JAK1* overexpression may have a bleak prognosis. Moreover, diverse immune infiltration cells and gene sets were positively correlated with *JAK1* expression level. Hence, to the best of our knowledge, our study is the first to reveal the potential mechanism by which *JAK1* functions in the TME and acts as a prognostic biomarker of NSCLC.

The TME plays a crucial role in the gene expression and clinical efficacy of tumour tissues, which are prerequisites and guarantees tumour immune escape [27]. The TME refers to the sum of various immune-related factors, mainly consisting of immune cells and immune-related molecules. In our study, we found that *JAK1* expression was significantly positively correlated with the infiltration of various immune cells (monocytes, neutrophils, B cells, dendritic cells, TAMs) in LUAD and LUSC. Presently, the antitumour function of manifold cells has been extensively recognized, especially CD8^+^ T cells [28], whose number reflects the immune system's ability to kill tumour cells to some extent. Moreover, CD8^+^ T cell density was positively correlated with the efficacy of immune checkpoint inhibitors (ICIs) in NSCLC and melanoma [29, 30]. This finding may provide an early indication for the efficacy of immunotherapy for NSCLC.

Another significant part of our study is that diverse gene sets were positively correlated with *JAK1* expression levels. First, M1 macrophage-related gene markers, such as *IRF5* and *NOS2*, and the gene marker *CD163* of M2 macrophages were strongly correlated with *JAK1* expression. These findings suggested that *JAK1* may play a role in regulating TAM polarization in the TME. Second, overexpression of *JAK1* is associated with a variety of T helper cells (Th1, Th2). This intense correlation may indicate that *JAK1* regulates T cell function in the immune microenvironment of NSCLC. Third, our study showed a significant correlation between Treg activation (*FOXP3*, *STAT5B*, *TGFB1*, *CCR8*, *CD25* in LUAD and LUSC) and induced T cell exhaustion (*PD-1*, *CTLA-4*, *TIM-3* in LUAD and LUSC) and *JAK1* overexpression. *PD-1* (programmed death receptor 1) is a vital immunosuppressive molecule expressed on the surface of T cells that regulates the immune system and promotes tolerance by downregulating the immune system’s response to human cells and by suppressing the inflammatory activity of T cells [31]. Additionally, *CTLA-4* and *Tim-3* are expressed on regulatory T cells and exhausted T cells as crucial receptor proteins, respectively [32, 33], and both are significantly positively correlated with *JAK1* expression. These results suggest that *JAK1* plays a potential role in recruiting immune-infiltrating cells in the TME of NSCLC.

Recent studies provide possible mechanisms which explains why *JAK1* overexpression correlates with immune infiltration and superior prognosis. Previous studies have shown that *JAK1* overexpression can lead to the activation of downstream interferon-stimulated genes, which can eventually exert a range of antitumour effects [34, 35]. These include increased antigen presentation by inducing proteasome subunits, activating transporters associated with antigen processing (TAP), stimulating major histocompatibility complex (MHC) molecules to be involved in antigen recognition and promoting chemokine production to exploit a first-hand antitumour role [36]. Remarkably, numerous studies have revealed that loss-of-function *JAK1* mutations are insinuative of immune evasion [11, 37, 38]. Research by Shin et al. [35] showed that *JAK1* mutations could induce primary resistance to *PD-1* inhibitors in melanoma and colon cancer patients. Rodig et al. [39] also indicated that loss of *JAK1* caused perinatal death in mice. Luo et al. [40] have shown that the response of melanoma to *PD-L1* inhibitor immunotherapy requires *JAK1* signaling, which may be related to its potentiated IFN-γ response in vivo and in vitro. Besides, researchers also point out that human melanoma cell lines are insensitive to interferon (IFN)-induced antitumor effects after *JAK1/2* knockout [41]. Consequently, *JAK1* may regulate immune-related pathways that affect the prognosis and immune infiltrates of NSCLC. Concrete mechanisms have yet to be explored.

However, the shortcomings of our descriptive study should be noted. First, the sequencing data and tumour tissue chips are based on a variety of platforms and databases, and systematic errors and bias are inevitable. Second, our study analysed only *JAK1* expression and immune cell infiltration using a variety of databases, which still needs to be verified by specific in vitro experiments. Finally, the precise regulatory pathway of *JAK1* in the TME of NSCLC still needs to be further explored.

In summary, the elevated expression of *JAK1* is associated with superior prognosis and abundant immune cell infiltration in NSCLC. These findings may lay the foundation for immunotherapy for NSCLC.

## Supplementary Information

**Additional file 1: Table S1.** Association between JAK1 expression and prognosis with different clinicopathological features of NSCLC by Kaplan-Meier plotter (specific survival data). **Fig. S1.** Correlation between JAK1 expression level and immune cell infiltration in LUSC from the TISIDB web portal (501 samples). (A-H) JAK1 expression had no significant correlation with infiltrating levels of Act_CD4 and was significantly positively correlated with infiltrating levels of Act_DCs, iDCs, neutrophils, NK cells, pDCs, Tcm_CD4 and Tem_CD8. LUSC, lung squamous cell carcinoma; Act_CD4, activated CD4 T cells; Act_DCs, activated dendritic cells; iDCs, immature dendritic cells; NK cells, natural killer cells; pDCs, plasmacytoid dendritic cells; Tcm_CD4, central memory CD4 cells; Tem_CD8, effector memory CD8 cells. A P value less than 0.05 indicated statistical significance.

## Data Availability

All data generated or analysed during this study are included in this article.
