# Supplementary material for: The expression landscape of JAK1 and its potential as a biomarker for prognosis and immune infiltrates in NSCLC
Source: BMC Bioinformatics. 2021 Sep 29;22:471. doi: 10.1186/s12859-021-04379-y (PMC8482691; doi:10.1186/s12859-021-04379-y)
Supplement: Supplementary file 1 — Additional file 1: Table S1. Association between JAK1 expression and prognosis with different clinicopathological features of NSCLC by Kaplan-Meier plotter (specific survival data). Fig. S1. Correlation between JAK1 expression level and immune cell infiltration in LUSC from the TISIDB web portal (501 samples). (A-H) JAK1 expression had no significant correlation with infiltrating levels of Act_CD4 and was significantly positively correlated with infiltrating levels of Act_DCs, iDCs, neutrophils, NK cells, pDCs, Tcm_CD4 and Tem_CD8. LUSC, lung squamous cell carcinoma; Act_CD4, activated CD4 T cells; Act_DCs, activated dendritic cells; iDCs, immature dendritic cells; NK cells, natural killer cells; pDCs, plasmacytoid dendritic cells; Tcm_CD4, central memory CD4 cells; Tem_CD8, effector memory CD8 cells. A P value less than 0.05 indicated statistical significance. [file 12859_2021_4379_MOESM1_ESM.doc]

**Table S1** Association between *JAK1* expression and prognosis with different clinicopathological features of NSCLC by Kaplan-Meier plotter (specific survival data)

| **Clinicopathological characteristics** | **mOS(month)** | | |  | **mPFS(month)** | | |
| --- | --- | --- | --- | --- | --- | --- | --- |
|  | JAK1 mRNA high | JAK1 mRNA low | *P*-value |  | JAK1 mRNA high | JAK1 mRNA low | *P*-value |
| **Gender** |  |  |  |  |  |  |  |
| male | 78 | 45 | **2.20E-05** |  | 34.76 | 12.39 | **0.0043** |
| female | 128.57 | 110.27 | 0.38 |  | 45.3 | 30.83 | 0.25 |
| **Stage** |  |  |  |  |  |  |  |
| 1 | 93 | 136.33 | **0.02** |  | 44 | 56.87 | 0.31 |
| 2 | 67 | 68.67 | 0.95 |  | 63 | 36.17 | 0.108 |
| 3 | 24.09 | 14.93 | 0.77 |  | - | - | - |
| **AJCC Stage T** |  |  |  |  |  |  |  |
| 1 | 179 | 104 | 0.44 |  | - | - | 0.94 |
| 2 | 67 | 52 | 0.44 |  | 19 | 52 | 0.073 |
| 3 | 21 | 20 | 0.65 |  | - | - | - |
| 4 | 12 | 17 | 0.52 |  | - | - | - |
| **AJCC Stage N** |  |  |  |  |  |  |  |
| 0 | 99 | 89 | 0.38 |  | - | - | **0.029** |
| 1 | 67 | 34 | 0.09 |  | 21 | 45 | 0.96 |
| 2 | 28 | 11 | **0.016** |  | - | - | - |
| **AJCC Stage M** |  |  |  |  |  |  |  |
| 0 | 78 | 57 | **0.013** |  | 25 | 59 | 0.07 |
| **Smoking history** |  |  |  |  |  |  |  |
| yes | 38.1 | 47.77 | **0.029** |  | 43 | 21.03 | 0.11 |
| no | - | - | 0.056 |  | 37 | 54.6 | 0.29 |
| **Chemotherapy** |  |  |  |  |  |  |  |
| yes | 42.38 | 40.21 | 0.74 |  | 34.76 | 40.21 | 0.74 |
| no | - | - | 0.075 |  | 50.56 | 36.17 | 0.44 |
|  |  |  |  |  |  |  |  |
|  |  |  |  |  |  |  |  |

**
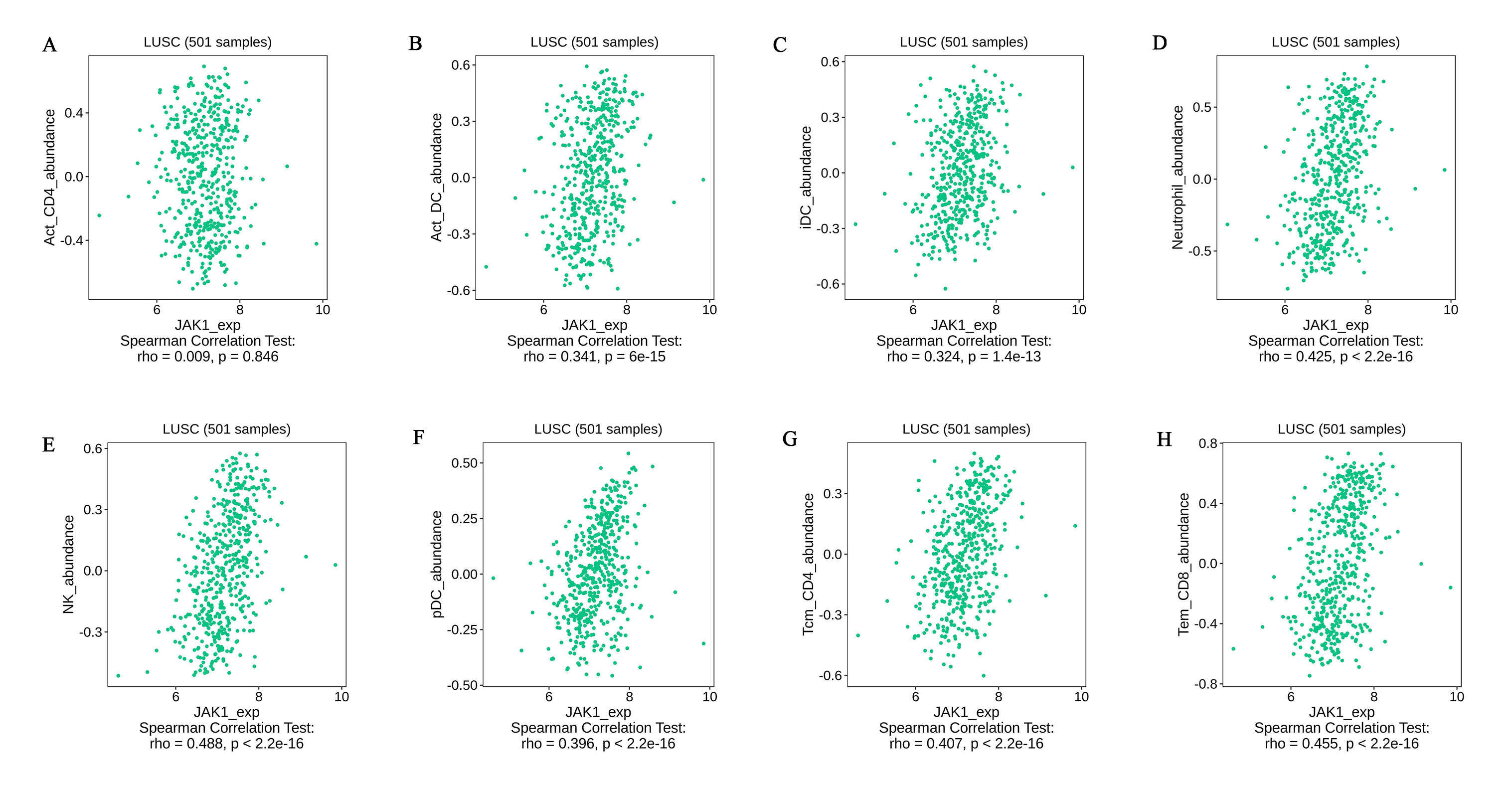
**

**Fig S1** Correlation between *JAK1* expression level and immune cell infiltration in LUSC from the TISIDB web portal (501 samples). (A-H) *JAK1* expression had no significant correlation with infiltrating levels of Act_CD4 and was significantly positively correlated with infiltrating levels of Act_DCs, iDCs, neutrophils, NK cells, pDCs, Tcm_CD4 and Tem_CD8. LUSC, lung squamous cell carcinoma; Act_CD4, activated CD4 T cells; Act_DCs, activated dendritic cells; iDCs, immature dendritic cells; NK cells, natural killer cells; pDCs, plasmacytoid dendritic cells; Tcm_CD4, central memory CD4 cells; Tem_CD8, effector memory CD8 cells. A *P* value less than 0.05 indicated statistical significance.
